# Right-side cardiac metastasis of squamous cell carcinoma from oesophagogastric junction cancer

**DOI:** 10.1093/ehjcr/ytae294

**Published:** 2024-06-12

**Authors:** Hiroyuki Matsumoto, Shun Ijuin, Toshihiko Terashi

**Affiliations:** Department of Cardiovascular Medicine, National Hospital Organisation Kagoshima Medical Center: Kokuritsu Byoin Kiko Kagoshima Iryo Center, 8-1, Shiroyama-cho, Kagoshima-shi, Kagoshima 892-0853, Japan; Department of Cardiovascular Medicine, National Hospital Organisation Kagoshima Medical Center: Kokuritsu Byoin Kiko Kagoshima Iryo Center, 8-1, Shiroyama-cho, Kagoshima-shi, Kagoshima 892-0853, Japan; Department of Cardiovascular Medicine, Kirishima Medical Center, Hayatocho-Matsunaga 3320, Kirishima-shi, Kagoshima 899-5112, Japan

**Figure:**
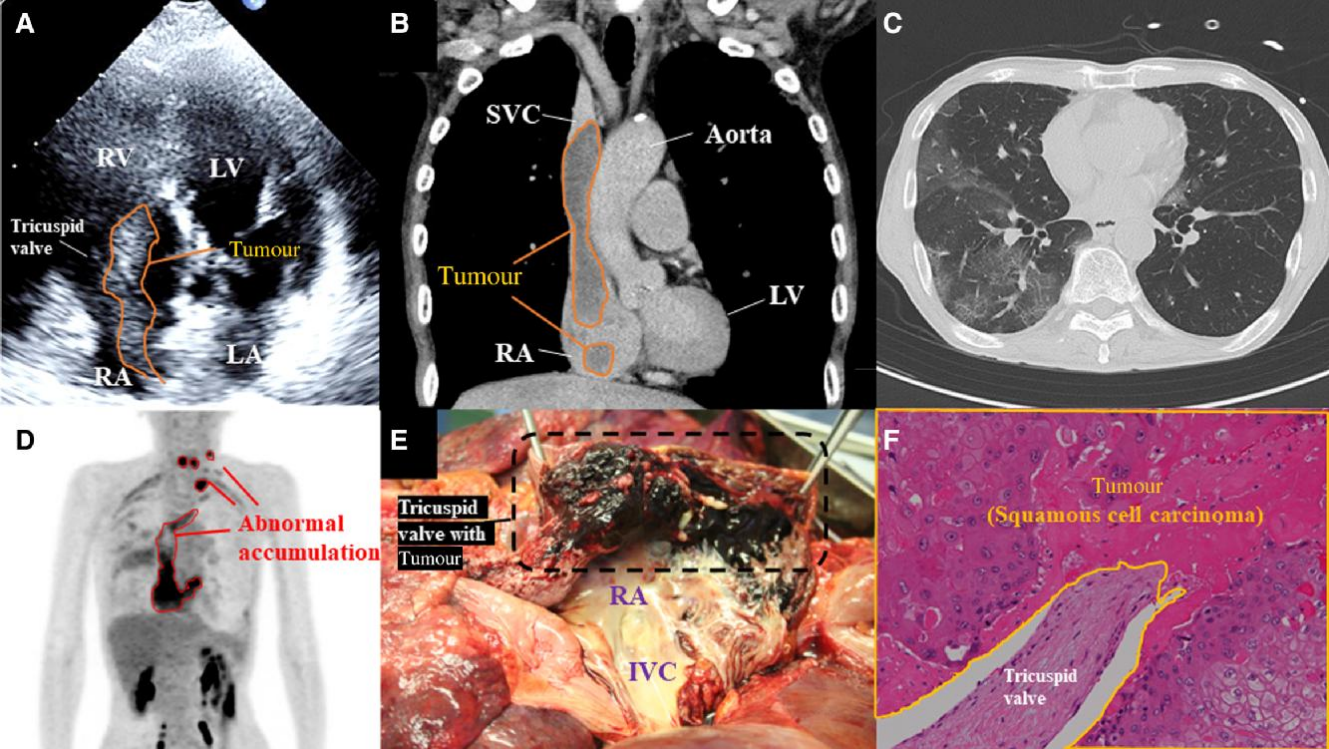


A 74-year-old man was transferred to our hospital for treatment of an intravascular embolus extending from the subclavian vein to the right atrium: that was not dissolved despite 3 months of oral anticoagulant therapy. He had been performed resection of oesophagogastric junction cancer 5 years ago and subsequently undergone post-operative chemotherapy. Although he was asymptomatic, the embolus was incidentally discovered on a follow-up computed tomography (CT). Echocardiography revealed a mobile high-intensity echo extending from the superior vena cava to the right ventricle, which was attached to the tricuspid valve (*Panel A*; Supplementary material online, *Video S1*). Contrast-enhanced CT demonstrated the continuous embolus from the left subclavian vein to the right ventricle (*Panel B*; Supplementary material online, *Video S2*), and diffuse ground-glass opacities were observed in the lung field, suggesting embolization in peripheral arteries of the lungs (*Panel C*). Fluorodeoxyglucose-positron emission tomography demonstrated that abnormal accumulation in the left supraclavicular fossa and cervical lymph nodes and from the left subclavian vein to the right ventricle (*Panel D;*Supplementary material online, *Video S3*), and we diagnosed as tumour embolization. We discussed treatment plans with surgeons. However, a highly invasive surgery involving removal of the left clavicle and subclavian vein was required, and he requested palliative care. Home oxygen therapy was introduced, and he was discharged home. He died 9 days after being discharged. Pathological autopsy revealed tumour embolus extending from the left internal jugular vein to the left and right pulmonary arteries, and histologically, the tumour had characteristics of squamous cell carcinoma (*Panels E* and *F*). This case is a rare case of squamous cell carcinoma in which cancer of the oesophagogastric junction metastasized to right-side cardiac chamber. Fluorodeoxyglucose-positron emission tomography was feasible to differentiate between thrombus and tumours.

## Supplementary Material

ytae294_Supplementary_Data

## Data Availability

The data underlying this article are available in the article and in its online Supplementary material.

